# Association of the *NOTCH4* Gene Polymorphism rs204993 with Schizophrenia in the Chinese Han Population

**DOI:** 10.1155/2015/408096

**Published:** 2015-10-28

**Authors:** Bao Zhang, Qian Rui Fan, Wen Hao Li, Ning Lu, Dong Ke Fu, Yan Jie Kang, Na Wang, Teng Li, Xiao Peng Wen, Da Xu Li

**Affiliations:** ^1^College of Medicine & Forensic, Health Science Center, Xi'an Jiaotong University, Xi'an 710061, China; ^2^Department of Food and Biological Engineering, Henan Industry & Trade Vocational College, Henan, Zhengzhou 451191, China; ^3^Department of Thoracic Surgery, The First Affiliated Hospital, Xi'an Jiaotong University, Xi'an 710061, China; ^4^Department of Stomatology, The First Affiliated Hospital, Xi'an Jiaotong University, Xi'an 710061, China

## Abstract

NOTCH4 regulates signaling pathways associated with neuronal maturation, a process involved in the development and patterning of the central nervous system. The *NOTCH4* gene has also been identified as a possible susceptibility gene for schizophrenia (SCZ). The objective of this study was to examine the relationship between *NOTCH4 *polymorphisms and SCZ in the Chinese Han population. The rs2071287 and rs204993 polymorphisms of the *NOTCH4* gene were analyzed in 443 patients with SCZ and 628 controls of Han Chinese descent. Single SNP allele-, genotype-, and gender-specific associations were analyzed using different models (i.e., additive, dominant, and recessive models). This association study revealed that the rs204993 polymorphism is significantly associated with susceptibility for SCZ and that the AA genotype of rs204993 is associated with a higher risk for SCZ (*P* = 0.027; OR = 1.460; 95% CI, 1.043–2.054). Our data are consistent with those obtained in previous studies that suggested that rs204993 is associated with SCZ and that the AA genotype of rs204993 demonstrates a higher risk. Further large-scale association analyses in Han Chinese populations are warranted.

## 1. Introduction

Schizophrenia (SCZ) is a chronic, severe psychotic mental disorder characterized by both positive (e.g., delusions, hallucinations, and thought disorders) and negative symptoms (e.g., social withdrawal, apathy, and cognitive impairment) [1]. As a serious neurological disability, SCZ affects approximately 1% of the general population worldwide and is regarded as a major public health problem, ranking ninth in terms of global disease burden [2]. Based on evidence from family-based, twin and adoption studies, which have implicated numerous genes in the etiology of SCZ, the disorder is currently understood as a polygenic neurodevelopment disorder caused by the interplay between environmental factors and genetics [3]. Although genome-wide association studies (GWAS) and candidate gene approaches to SCZ have produced many positive results [4, 5], only a small proportion of the genes have been consistently studied across multiple different populations. Therefore, the exploration of the true etiology and genetic mechanisms underlying the associations between genes and SCZ in various genetically independent populations is necessary.

The* NOTCH4* (neurogenic locus notch homolog protein 4) gene, located at the centromeric end of the HLA class III region, has been implicated in SCZ based on evidence from several genetic studies [6]. Some evidence has suggested that* NOTCH4 *polymorphisms, including microsatellite and SNPs (e.g., rs3132935, rs3131296, and rs3809842), have been described [7–10]. More recently, a GWAS study identified rs2071287 of the* NOTCH4* gene as the most statistically significant marker, which was confirmed through a follow-up analysis in a Japanese population [11]. Furthermore, rs2071287 and rs3131296, which display very strong LD with each other, were identified as the SNPs that are most likely to be functionally relevant to SCZ etiology, suggesting that it is necessary to determine the association of rs2071287 with SCZ [12, 13]. Another SNP, rs204993, was found to be significantly associated with SCZ through a family-based association study of 218 Taiwan nuclear families [14]. To date, few studies have investigated the association of* NOTCH4* with SCZ in the Chinese population, particularly regarding the SNPs rs2071287 and rs204993. Moreover, a genetic model describing the importance of the* NOTCH4 *gene in SCZ has not yet been elucidated.

Therefore, we performed a case-control study to examine the possibility of* NOTCH4 *polymorphisms being associated with SCZ in the Chinese Han population and to determine the inheritance models for the association study.

## 2. Materials and Methods

### 2.1. Subjects

The case-control samples included 443 SCZ cases (224 females and 219 males, mean age: 36.1 ± 10.2 years) and 628 healthy controls (358 females and 270 males, mean age: 35.7 ± 9.7 years). The subjects were all original northern Han Chinese recruited from The First Affiliated Hospital of Xi'an Jiaotong University. The available information (i.e., personal history, hospital record, and family-history report) was analyzed based on The Fourth Edition of the Diagnostic and Statistical Manual of Mental Disorders (DSM-IV) to obtain a consensus diagnosis agreed on by at least two experienced senior psychiatrists. After undergoing physical examinations, subjects with substance-induced psychotic disorders, learning disabilities, head injuries, and other symptomatic psychoses were excluded from the present study. Moreover, the controls were confirmed to not have any mental illness and were matched with the patients in age, sex, and educational level. Informed consent was obtained from all of the participants. The study was approved by the Xi'an Jiaotong University School of Medicine and The First Affiliated Hospital of Xi'an Jiaotong University.

### 2.2. SNP Genotyping

Two SNPs, rs2071287 and rs204993, showed significant associations with SCZ in previous manuscripts [11, 14]. They were located in the intron and 3′ untranslated region of the* NOTCH4* gene and detected in the present study. Genomic DNA was extracted from the peripheral blood leukocytes of all of the subjects based on the manufacturer's recommendations (Omega, Bio-tek, GA, USA). All of the SNPs were genotyped by the SNPscan technique (Genesky Biotechnologies Inc., Shanghai, China) according to a previous study [15]. Five percent of the samples with high DNA quality were randomly repeated to guarantee the genotyping quality. The average genotype call rate for all of the markers was 96.5%.

### 2.3. Statistical Analysis

The G^*∗*^Power program was used to calculate the statistical power of our sample size according to Cohen's method [16]. The sample size exhibited >80% power for the detection of significant (*P* < 0.05) associations at an effect size index of 0.1 (corresponding to a “weak” gene effect).

Hardy-Weinberg equilibrium (HWE) was detected for the SNP genotypes by the Chi-square test, which was also used to calculate the differences in the genotype and allele frequencies between the cases and controls. We performed logistic regression analyses to identify SCZ-associated SNPs by their odds ratios (OR), 95% confidence intervals (CIs), and corresponding *P* values. Furthermore, single SNP analyses were performed using multiple inheritance models, namely, additive, dominant, and recessive models, similarly to an earlier study [15]. Moreover, stratified analyses were conducted to detect whether differences in gender and genetics influenced such associations. All of the statistical analyses were conducted using PLINK (version 1.07). Differences were considered significant when the *P* value was 0.05.

## 3. Results

For the study, 443 eligible individuals with schizophrenia and 628 healthy controls were enrolled. Two SNPs (rs2071287 and rs204993) in the* NOCTH4* gene were genotyped and analysed.

### 3.1. Hardy-Weinberg Equilibria of Two Variants

None of the rs2071287 or rs204993 SNPs showed deviation from Hardy-Weinberg equilibria in the control population (*P* = 0.18, and *P* = 0.79, resp.).

### 3.2. Association with the Two Variants

The allele and genotype frequencies of the two SNPs (rs2071287 and rs204993) are shown in Table 1. The estimated risks associated with these polymorphisms in the SCZ patients and the healthy controls were tested according to three models (i.e., dominant, recessive, and addictive models), also illustrated in Table 1. Considering all the subjects, the allele frequencies of rs2071287 and rs204993 for the patients with SCZ were not significantly different from those of the control group. However, notably, genotype association analysis for rs204993 suggested a significant association with SCZ under a recessive model, and the AA genotype of rs204993 was associated with a higher risk of SCZ (*P* = 0.027; OR = 1.460; 95% CI, 1.043–2.054).

### 3.3. Association with the Two Variants of the Gender-Specific Analyses

To examine whether gender would play a role in the association, we analyzed our data by separating males and females on the basis of results above (Table 2). Furthermore, we found that rs204993 showed marginally significant genotypic (*P* = 0.084; OR = 1.561; 95% CI, 0.939–2.596) associations with SCZ in males, but not in females.

## 4. Discussion

The* NOTCH4* gene located on 6p21.3 encodes a member of the NOTCH family, the members of which play a pivotal role in deciding cell fate, particularly during the neurodevelopmental process to promote proliferative signaling [14, 17–19]. In particular, NOTCH4 regulates the signaling of the maturation of neurons and glia from neural stem cells [13, 20, 21]. As an important neurodevelopment-related gene, transcripts of* NOTCH4* can be detected in the developing nervous system [22], which makes* NOTCH4* a potential candidate gene for neurodevelopment disorders, such as SCZ [23]. Previous studies identified* NOTCH4* polymorphisms implicated in the risk of SCZ. In this study, two SNPs of the* NOTCH4* gene were detected to confirm their association with SCZ in the Han Chinese population. We found that the allele and genotype distributions of rs2071287 demonstrated insignificant associations. Notably, the genotype distribution of the rs204993 polymorphism demonstrated a significant association with SCZ under a recessive model (*P* = 0.027).

However, a previous GWAS study, as well as a follow-up analysis, revealed that rs2071287 was associated with SCZ in the Japanese population [11, 24]. In contrast, our results are consistent with other studies that reported an insignificant association between the rs2071287 polymorphism and SCZ [25, 26]. The rs2071287 frequency of the “A” allele differs considerably (0.31 for Japanese, 0.36 for Bulgarian, and 0.38 for Chinese Han), which suggests that subjects from different ethnic backgrounds may exhibit SCZ-related genetic heterogeneity. Hence, our findings suggest that this SNP may represent a risk for SCZ under certain ethnic backgrounds rather than universally.

A family-based association study preliminarily showed an association between rs204993 and SCZ. Consistent with this finding, the present case-control study suggested that* NOTCH4* rs204993 is significantly associated with SCZ. The rs204993 polymorphism of the* NOTCH4* gene is located in the 3′UTR region. And rs204993 “A” allele is the major allele in the study population, suggesting this polymorphism may be in LD with another potentially functional SNP that affects NOTCH4 expression. Furthermore, rs204993 has also been mapped to the adjacent gene PBX2, which is also located within the SCZ-related region in the major histocompatibility complex (MHC) [27].

Based on the available findings, because family-based and case-control association studies have demonstrated significant associations between rs204993 and SCZ, we can only predict that rs204993 associates with SCZ as it is in LD with another functional SNP which may have an effect on gene expression. We hypothesize that further functional exploration (such as* in vitro* and* in vivo *experimentation) of this polymorphism could lead to an important understanding of the genetic pathophysiology of schizophrenia.

This study has several limitations that need to be addressed. First, the relatively small sample size of our experimental study limits the interpretation of the results for the Chinese Han population. However, the observation of a statistically significant association is encouraging. Furthermore, this study was performed at a single center, which may potentially limit the generalizability of the findings. Moreover, other SNPs in the candidate region/gene in addition to the reported risk-related SNP may account for the association with schizophrenia detected in different populations. Despite these limitations, our current study provides useful data for a future meta-analysis of these psychosis markers.

In summary, our data support an association between* NOTCH4* and SCZ. However, further studies using larger numbers of samples of different ethnic groups should be performed to determine the validity of such relationships.

## Figures and Tables

**Table 1:** Analysis of the allele and genotype frequencies of a single SNP association.

Makers	Allele freq. (%)		*P* value^a^	OR (95% CI)	Genotype (*N*)			HWE *P* value	Model	OR (95% CI)	*P* value^a^
Rs204993	G	A			GG	AG	AA				
SCZ	41.3	58.7	0.223	1.115 (0.936–1.329)	78	210	155	0.182	Add	1.121 (0.937–1.342)	0.213
CTR	38.7	61.3			80	326	222		Dom	1.016 (0.788–1.311)	0.903
									Rec	1.464 (1.043–2.054)	***0.027***

Rs2071287	A	G			AA	AG	GG				
SCZ	38.0	62.0	0.530	1.059 (0.886–1.264)	68	201	174	0.794	Add	1.058 (0.887–1.263)	0.532
CTR	36.7	63.3			83	295	250		Dom	1.022 (0.797–1.311)	0.861
									Rec	1.191 (0.842–1.682)	0.324

SCZ: schizophrenia; CTR: control; CI: confidence interval; OR: odds ratio.

Significant *P* values and HWE *P* values are shown in italic bold font.

^a^ *P* values of the normal Chi-square statistics.

Add: addictive model; Dom: dominant model; Rec: recessive model.

**Table 2:** Analysis of sex-specific allele and genotype associations.

Markers and sex		Allele freq. (%)		*P* value^a^	OR (95% CI)	Genotype (*N*)			Model	OR (95% CI)	*P* value^a^
rs204993		G	A			GG	AG	AA			
F	SCZ	41.1	58.9	0.366	1.118 (0.878–1.422)	40	104	80	Add	1.120 (0.878–1.429)	0.362
	CTR	38.4	61.6			48	179	131	Dom	1.039 (0.734–1.471)	0.830
									Rec	3.242 (0.589–17.85)	0.145
M	SCZ	41.6	89.7	0.432	1.109 (0.857–1.433)	38	106	75	Add	1.118 (0.856–1.461)	0.414
	CTR	39.1	89.6			32	147	91	Dom	0.976 (0.670–1.422)	0.899
									Rec	1.561 (0.939–2.596)	0.084

rs2071287		T	C			TT	TC	CC			
F	SCZ	39.3	60.7	0.098	1.229 (0.962–1.569)	36	104	84	Add	1.222 (0.960–1.555)	0.104
	CTR	34.5	65.5			45	157	156	Dom	1.287 (0.915–1.812)	0.148
									Rec	1.332 (0.829–2.14)	0.236
M	SCZ	36.8	63.2	0.358	1.885 (0.683–1.148)	32	97	90	Add	0.884 (0.680–1.148)	0.355
	CTR	39.6	60.4			44	138	94	Dom	0.766 (0.530–1.106)	0.154
									Rec	1.045 (0.629–1.737)	0.866

SCZ: schizophrenia; CTR: control; CI: confidence interval; OR: odds ratio.

^a^ *P* values of the normal Chi-square statistics.

Add: addictive model; Dom: dominant model; Rec: recessive model.
